# Malleable nature of mRNA-protein compositional complementarity and its functional significance

**DOI:** 10.1093/nar/gkv166

**Published:** 2015-03-08

**Authors:** Mario Hlevnjak, Bojan Zagrovic

**Affiliations:** Department of Structural and Computational Biology, Max F. Perutz Laboratories, University of Vienna, Campus Vienna Biocenter 5, 1030 Vienna, Austria

## Abstract

It has recently been demonstrated that nucleobase-density profiles of typical mRNA coding sequences exhibit a complementary relationship with nucleobase-interaction propensity profiles of their cognate protein sequences. This finding supports the idea that the genetic code developed in response to direct binding interactions between amino acids and appropriate nucleobases, but also suggests that present-day mRNAs and their cognate proteins may be physicochemically complementary to each other and bind. Here, we computationally recode complete *Methanocaldococcus jannaschii, Escherichia coli* and *Homo sapiens* mRNA transcriptomes and analyze how much complementary matching of synonymous mRNAs can vary, while keeping protein sequences fixed. We show that for most proteins there exist cognate mRNAs that improve, but also significantly worsen the level of native matching (e.g. by 1.8 viz. 7.6 standard deviations on average for *H. sapiens*, respectively), with the least malleable proteins in this sense being strongly enriched in nuclear localization and DNA-binding functions. Even so, we show that the majority of recodings for most proteins result in pronounced complementarity. Our results suggest that the genetic code was designed for favorable, yet tunable compositional complementarity between mRNAs and their cognate proteins, supporting the hypothesis that the interactions between the two were an important defining element behind the code's origin.

## INTRODUCTION

The relationship between mRNAs and their cognate proteins, as defined by the universal genetic code, is a cornerstone of all known biological systems. The origin of the code, however, remains largely unexplained, representing one of the most important foundational problems in molecular biology that are still open. Recently, we have demonstrated a remarkable degree of proteome-wide compositional complementarity between mRNAs and their cognate proteins. This has provided not only an important clue as to the code's origin, but also suggested that the relationship between the two biopolymers could extend beyond just unidirectional information transfer (1–3). More specifically, we have uncovered a strong correlation between the pyrimidine content of individual mRNA coding sequences and the average propensity of their cognate protein sequences to interact with pyrimidine mimetics. The latter property of protein sequences was derived from the so-called polar requirement (PR) scale, which captures the propensity of individual amino acids to interact with substituted pyridines, such as dimethylpyridine (4,5). Importantly, we have shown for a number of different proteomes that pyrimidine density profiles of mRNA coding sequences closely match the PR profiles of their cognate protein sequences (1). Briefly put, mRNA regions that are rich in pyrimidines code for protein regions that exhibit high propensity to interact with pyrimidine mimetics, and *vice versa*.

We have also derived knowledge-based interaction propensities between individual RNA nucleobases and amino-acid residues from contact statistics in a large set of high-resolution 3D structures of RNA-protein complexes (2,3). Moreover, we have used classical molecular dynamics simulations and free energy techniques to provide a detailed, physically realistic picture of nucleobase/amino-acid interactions at the atomistic level (6,7). This has allowed us to not only confirm the above findings using orthogonal approaches, but also extend them to the case of purines and especially guanine. Adenine-rich mRNA stretches, interestingly, exhibit the opposite behavior in that they tend to code for protein stretches with an aversion for interacting with adenines. Taken together, the above observations provide strong support for the idea that the code originated as a consequence of direct binding preferences between amino acids and their cognate codons (4,8–9), especially those which are adenine-poor. Adenine-rich codons, in turn, may have entered the code in order to modulate and weaken this binding (2). Importantly, however, our results suggest that appreciable signatures of binding can only be seen if one examines relatively long, unstructured mRNA and protein stretches (1–3,6–7). Even more significantly, our results give rise to a novel, potentially far-reaching hypothesis that even present-day mRNAs and their cognate proteins might in general be physicochemically complementary to each other and bind, especially in adenine-poor mRNA regions (1–3,6–7,10).

An important question to be addressed in this context concerns the extent to which the observed compositional complementarity can be modulated (i.e. its malleability), while still observing the prescriptions of the universal genetic code. In other words, does the code allow for markedly different levels of complementarity between a given protein sequence and various synonymous mRNA sequences that could potentially code for it? Moreover, how optimal is the level of complementarity exhibited by native mRNA sequences? The fact that an amino acid can be encoded by more than one codon essentially allows one to rewrite the mRNA coding sequence, corresponding to one and the same protein sequence, in many different ways. Preferential use of particular codons as opposed to their expected occurrences based on the universal genetic code, the codon-usage bias, generally differs between different organisms and different genes (11,12). How does the fact that some amino acids are encoded by multiple codons affect the relationship between mRNA nucleobase content and nucleobase-interaction propensities of their cognate protein sequences?

Here, we address these questions by focusing on the relationship between the pyrimidine content of mRNA sequences and the PR of their cognate protein sequences. There are nine amino acids encoded by codons with varying pyrimidine content (Leu, Ile, Val, Pro, Thr, Ala, Gly, Ser and Arg), which together occupy a major fraction of the genetic code table (41 out of a total of 61 non-stop codons). In the present analysis, we recode mRNA sequences by varying the pyrimidine content of the codons corresponding to these nine amino acids and evaluate the extent to which this modulates the observed correlations with protein PR, which in turn does not change. Effectively, for three different organisms representing each of the three domains of life (*Methanocaldococcus jannaschii* for Archea, *Escherichia coli* for Bacteria and *Homo sapiens* for Eukarya), we explore the influence of codon usage on the relationship between pyrimidine content of mRNAs and PR profiles of their cognate protein sequences. In this way, we probe the limits of mRNA-protein compositional complementarity levels as set by a combination of the genetic code and various codon-usage patterns in recoded mRNAs. Finally, we should emphasize that the present study is primarily aimed at analyzing the properties of mRNA-protein compositional complementarity and not finding evidence concerning the hypothesis that such complementarity reflects an intrinsic potential of the two biopolymers to bind (1–3). While our results, as discussed below, do provide support for this still largely untested proposal, our principal focus here is the mRNA-protein compositional complementarity, which in contrast is a robust, easily reproducible fact.

## MATERIALS AND METHODS

### Data sets

Complete proteomes of *M. jannaschii, E. coli* and *H. sapiens* were extracted from the UniProtKB database (13) (June 2013 release), excluding proteins designated as ‘uncertain’ and retaining only the reviewed Swiss-Prot entries for downstream analysis. The starting sets contained 1782, 4157 and 19618 proteins for *M. jannaschii, E. coli* and *H. sapiens*, respectively. Where available, the coding sequences (CDS) of their corresponding mRNAs were downloaded from The European Nucleotide Archive (ENA) (14) using the ‘Cross-references’ section of each UniProtKB entry, while ensuring that ENA-translated mRNAs match the corresponding UniProtKB canonical protein sequences perfectly. Protein and mRNA sequences with non-canonical amino acids or nucleotides were excluded from the analysis. This resulted in 1667, 4149 and 14419 protein-mRNA pairs for *M. jannaschii, E. coli* and *H. sapiens* proteomes, respectively.

### Profile-matching calculations

For sequence-profile correlations, a sliding window-averaging procedure was used (window of 21 for proteins and 63 for mRNAs), with all protein sequences shorter than or equal to twice the window size (42 residues) excluded. We have already shown that correlations exhibit only a weak dependence on the size of the averaging window—here, we have chosen the window size that was used in previous studies (1–3,6–7,10). Our final sets contained 1666, 4091 and 14413 protein-mRNA pairs for *M. jannaschii, E. coli* and *H. sapiens*, respectively. Protein PR profiles were calculated using the computationally derived PR scale (5), as previously described (1), while the level of complementarity between window-averaged mRNA pyrimidine content profiles and protein PR profiles was quantified using Pearson correlation coefficients *R*.

### mRNA recoding

**Figure 1.**
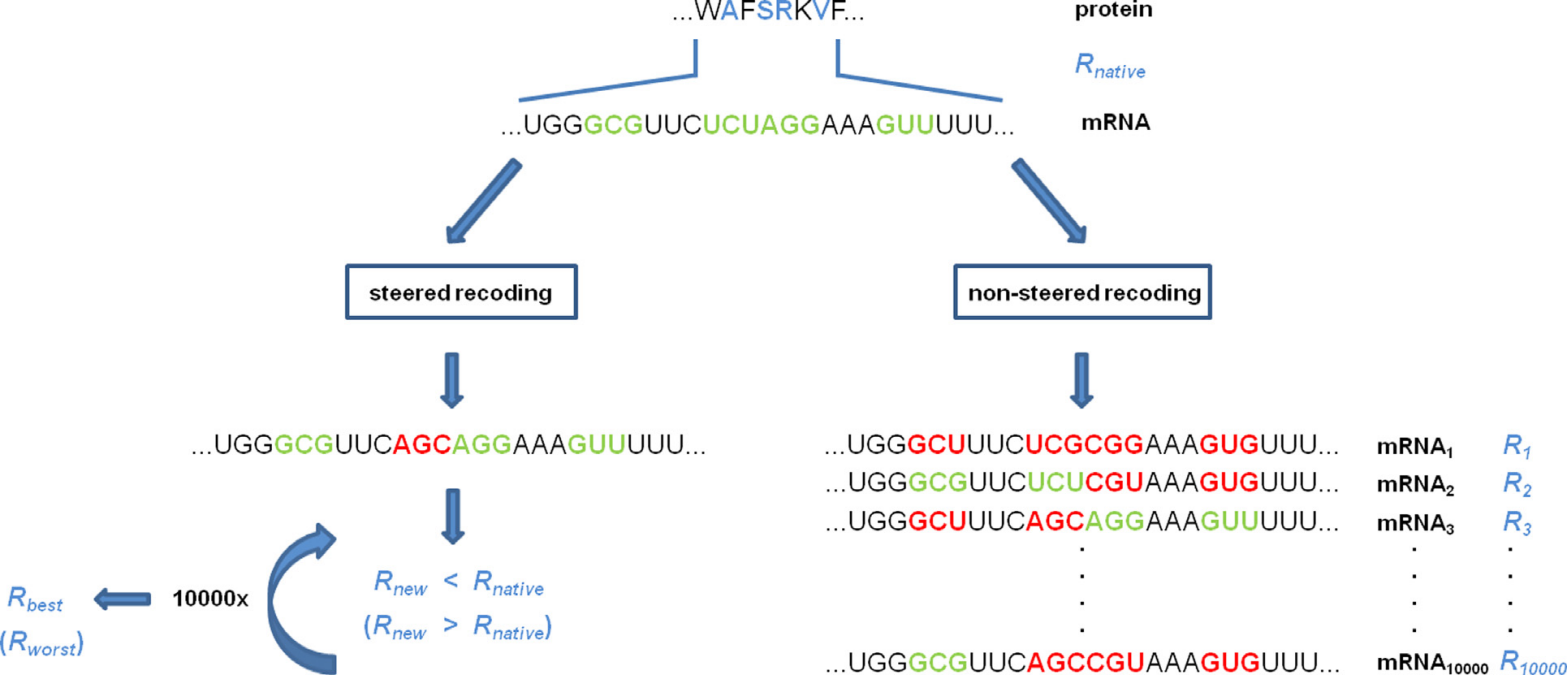
A schematic of the recoding procedure. We recode native mRNA sequences by varying the pyrimidine content of the codons corresponding to Leu, Ile, Val, Thr, Ala, Pro, Gly, Ser or Arg, while protein sequences do not change. Amino acids with codons whose pyrimidine content can vary are shown in blue, and their corresponding codons in green when native, or red when recoded. In steered recoding (left), we randomly change one codon in each out of 10 000 cycles, evaluate the complementarity by using the Pearson correlation coefficient (*R*_new_) and accept the change if it goes in the desired direction or reject it if not, finally optimizing mRNA-protein matching (*R*_best_) or mismatching (*R*_worst_). In non-steered recoding (right), we recode native mRNAs independently 10 000 times without optimizing the native level of matching (*R*_native_), resulting in a range of *R*s (*R_1_, R_2_*, …, *R_10000_*) for each native mRNA.

Two types of recoding procedures were used for sampling the mRNA sequence space: steered and non-steered. In steered recoding, each mRNA sequence went through 10 000 steps of a Monte Carlo-type procedure in which at each step a single, randomly chosen codon was reassigned to another synonymous codon. Reassignments were carried out only at those positions at which choosing a synonymous codon could lead to a change in pyrimidine content, and were attempted following the frequency of a given codon type in the standard genetic code. In this way, each step of the Monte Carlo procedure resulted in a newly recoded mRNA, which then served as the input for the next cycle of recoding. Since in steered recoding the aim was to either increase or decrease the level of matching between a protein's PR profile and its cognate mRNA PYR profile as compared to the native mRNA, after each codon reassignment step the two profiles were compared by calculating the Pearson *R* between them. If the goal was to optimize matching and a given codon change resulted in improved matching, the change was accepted, and if not, the codon selection was repeated. Conversely, if the goal was to optimize mismatching, changes which increased the level of mismatching were selected and others rejected. In this way, steered recoding progressively either increased or decreased the level of profile-matching between an mRNA and its cognate protein, and resulted in a single best- or worst-matched mRNA for each protein in a given proteome (Figure 1, left). In non-steered recoding, on the other hand, each mRNA was recoded independently 10 000 times by each time randomly changing all degenerate codons that could lead to a change in pyrimidine content. In this type of recoding, the same native mRNA served as input for a new recoding cycle, finally resulting in a set of 10 000 independent, recoded mRNA variants per each native mRNA in the transcriptome (Figure 1, right).

Both randomization procedures were performed using a reduced alphabet (PUR or PYR) with codon selection proportional to the occurrence of a given codon in the standard genetic code. Leucine, for example, is specified by six codons in the genetic code table with four of them having the pyrimidine content of 66.7% and two of them having the pyrimidine content of 100%. This means that the probability of choosing these two codon types during recoding was 4/6 = 66.7% in the first case or 2/6 = 33.3% in the second. In general, depending on the relative frequencies of different codon types for a given amino acid, the probabilities of occurrence of individual codon types varied.

In the standard genetic code GUG, UUG and AUU encode Val, Leu and Ile, respectively, which are all among amino acids targeted by our recoding approaches. On the other hand, many non-eukaryotes, including *E. coli*, are known to use GUG, UUG or AUU codons for initiator Met (15,16), which in principle could be a source of noise in our analysis. In order to account for these alternative encodings systematically, all start codons were excluded from the recoding procedure, and were simply kept fixed.

### Functional enrichment and depletion analysis

The complete list of species-specific Gene Ontology (GO) annotation terms was obtained from the UniProt-GOA (17,18) and GO databases (19,20) (Dec 2013 release). For each organism, we first evaluated the distribution of differences (Δ*R*) between Pearson correlation coefficients corresponding to the worst- and the best-matched mRNAs of every protein in the proteome (i.e. its malleability as defined by Δ*R* = *R*_worst_ – *R*_best_). Subsequently, we compared the top and the bottom 5% of such a distribution against the full background proteome of the same organism. Here, we used the EASE modification (21,22) where for each obtained protein count corresponding to a given GO term in the subset, we subtracted 1 in the case of enrichment or added 1 in the case of depletion. This procedure makes the downstream statistical analysis more stringent by penalizing the significance of those GO terms with low protein counts (21). In this way, for each GO term present in a given organism, we obtained protein counts for the analyzed subset and the background proteome, allowing us to assess the significance of the observed enrichment or depletion of each GO term via Fisher's exact test (22–24). Finally, we derived the false discovery rate (FDR)-corrected one-sided *P*-values (24) for the enrichment or depletion using a significance cutoff of 0.01.

### Codon-usage distances

As a measure of the distance between codon-usage patterns in different contexts, we used root-mean-square deviation between codon occurrences defined as:

\begin{equation*} {\rm RMSD} = \sqrt {\sum\limits_i^{20} {\frac{{\left( {x_{i,j} - x_{i,k} } \right)^2 }}{{20}}} } \end{equation*}

where *j* and *k* represent different contexts in which fractions of a given codon *i* are being compared. For example, these could include codon occurrences in the standard genetic code or in the native or the best-matched mRNA transcriptome for a given species. Here, codon occurrences in the standard genetic code (or genetic-code-based patterns of codon usage) refer to the expected occurrences of individual codons as dictated by the universal genetic code table. Note that in the reduced PUR/PYR alphabet the genetic code shrinks to only 20 codons when considering only those amino acids which have degenerate codons in terms of PYR content (Leu, Ile, Val, Thr, Ala, Pro, Gly, Ser or Arg). For example, instead of six codons for serine (AGU, AGC, UCA, UCG, UCU, UCC), the reduced representation results in only three codons with either 1 (RRY), 2 (RYY) or 3 (YYY) pyrimidines, denoted here as Ser1, Ser2 and Ser3.

### Multidimensional scaling (MDS)

MDS in 3D was performed using a built-in R (version 2.14.1) function *cmdscale* on a pairwise distance matrix (with RMSD as defined above) of a set of 10 different codon-usage patterns (standard genetic code, 3 native, 3 best- and 3 worst-matched mRNA transcriptomes corresponding to each of the analyzed organisms).

## RESULTS

To what extent can one alter the level of matching between native mRNA pyrimidine-content profiles and their cognate proteins’ PR profiles if mRNAs are recoded using synonymous codons that vary in their pyrimidine content? In order to address this question, we have recoded each mRNA using a steered Monte Carlo procedure (see Methods for details) in which at each step we reassign a codon at a randomly chosen position to one of its synonymous codons at random, with the newly recoded mRNA serving as input for the next recoding step, as illustrated in Figure 1 (left). In one variant of this process, we only accept those reassignments for which the matching strictly improves, resulting ultimately in an optimally matched mRNA/protein pair. In another variant, we only accept those changes for which the matching strictly deteriorates, resulting ultimately in an optimally anti-matched mRNA/protein pair. Importantly, in all of mRNA recoding attempts, protein sequences remain unchanged.

**Figure 2.**
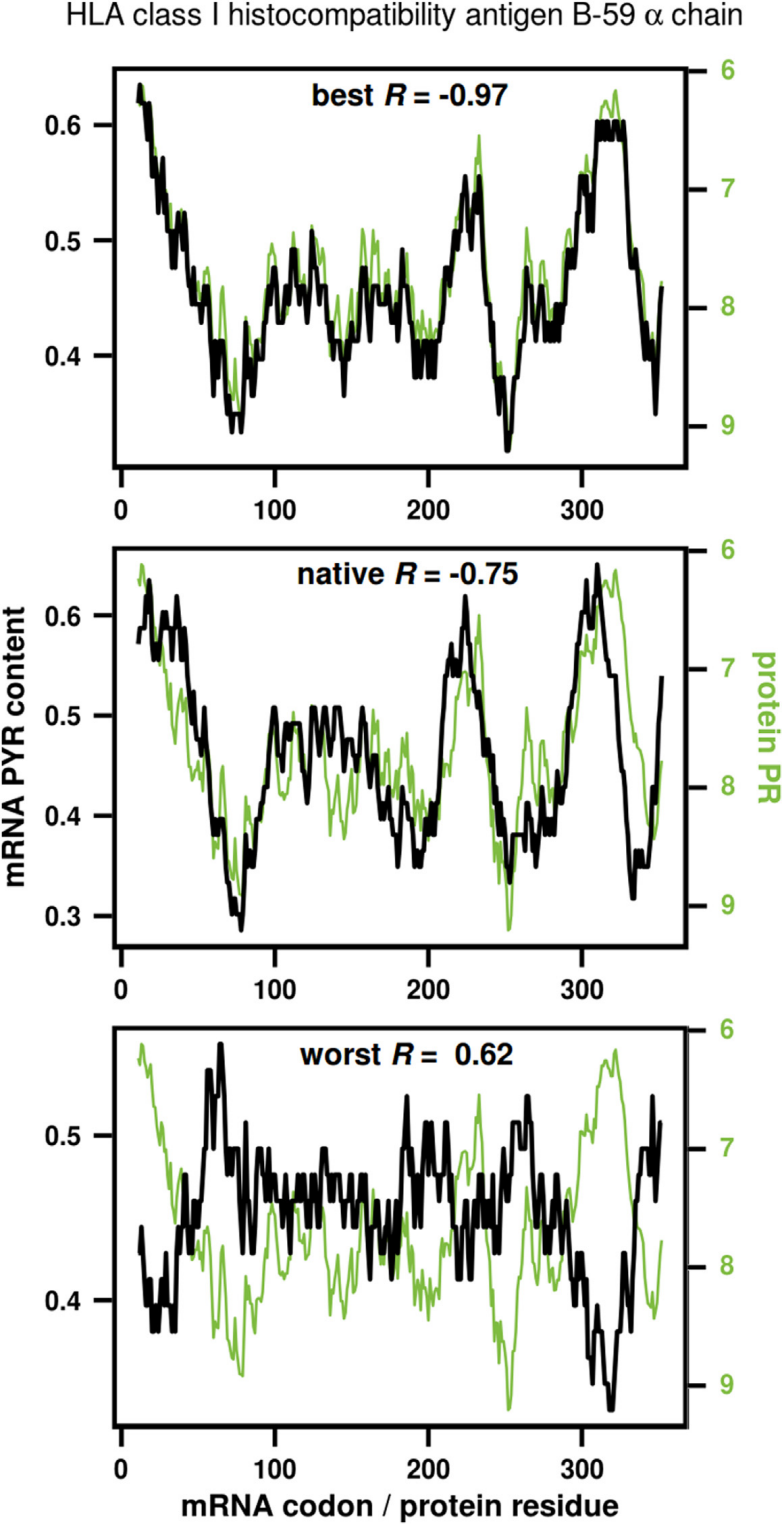
mRNA pyrimidine content (%PYR) and protein polar requirement (PR) profiles for human HLA class I histocompatibility antigen B-59 α chain (UniProt AC: Q29940). Shown are profiles for the best (top), native (middle) and worst (bottom) mRNA in terms of matching with the protein PR, as obtained by steered recoding.

In Figure 2, we show the results of this type of optimization for a representative human protein (HLA class I histocompatibility antigen B-59 α-chain), whose level of matching between its PR sequence profile and its native mRNA's PYR profile corresponds to the median over the complete human proteome (*R*_native_ = −0.75). Note that the PR scale is defined such that the lower the number, the higher the propensity to interact with pyrimidine mimetics. Therefore, negative values of the Pearson correlation coefficient correspond to direct matching between the mRNA PYR content and its cognate protein's propensity to interact with pyrimidine mimetics and *vice versa*. In Figure 2, we show the comparison between the protein and its native mRNA as well as the same for the recoded best- and worst-matched synonymous mRNAs (*R*_best_ = −0.97, top panel, and *R*_worst_ = 0.62, bottom panel, respectively). As can be seen, rewriting of the mRNA for this particular protein can result in an improvement in the level of matching of about 0.2 Pearson units (Figure 2, top) or its deterioration of well over 1 Pearson unit, effectively changing the sign of the correlation in the direction of anti-matching (Figure 2, bottom).

**Figure 3.**
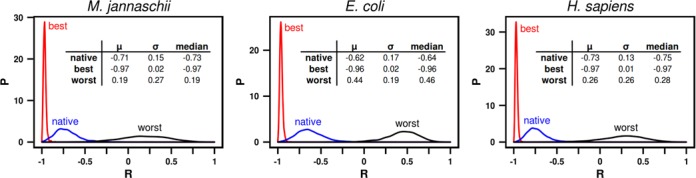
Profile-matching distributions for native transcriptomes and transcriptomes constructed from the best and the worst mRNAs in terms of profile matching. Shown are distributions obtained via steered recoding for *M. jannaschii, E. coli* and *H. sapiens*. Inset: distribution parameters (mean, sigma and median).

What do these changes look like at the level of complete transcriptomes? By collecting all of the best-matched and all of the worst-matched mRNAs obtained as a result of steered recoding, we can recreate full mRNA transcriptomes and examine their profile-matching distributions (Figure 3). Not unexpectedly, the distributions of Pearson *R*s, capturing the degree of matching, shift leftward for the best-matched recoded mRNAs and rightward for their worst-matched recoded counterparts. Importantly, however, the magnitude of this shift is substantial in both directions and for all organisms analyzed (Figure 3). For example, for the human proteome the median of the distribution decreases from its native value of −0.75 by 0.22 Pearson units in the case of the best-matching distribution or increases by a remarkable 1.03 Pearson units in the case of the worst-matching distribution, with similar changes seen for *M. jannaschii* and *E. coli* proteomes (Figure 3). In fact, the median of the *E. coli* worst-matching distribution shifts to 0.46, indicating not only a loss of matching, but actually a high level of anti-matching even for an average protein. In other words, regardless of the organism examined, it is possible to recode the set of native mRNAs to obtain a transcriptome that as a whole gives a significantly worse, but also significantly better level of profile matching as compared to the one observed for native mRNA sequences (Figure 3). In general, however, given the high level of matching with native sequences to begin with, the shifts in the direction of worse matching are significantly greater than those in the direction of better matching (e.g. 7.6 viz. 1.8 standard deviations on average for *H. sapiens*, respectively). Here it should also be mentioned that the level of sampling used in steered recoding appears to be sufficient to capture the main features of the resulting profile-matching distributions and in particular those with worst-matched transcripts: in Supplementary Figure S1, we show the average Pearson *R* as a function of the number of recoding steps for the *M. jannaschii* transcriptome where the distribution mean shifts from 0.19 to 0.22 upon an increase in the extent of recoding from 10^4^ to 10^5^ steps i.e. the improvement upon a 10-fold increase in sampling appears to be only marginal.

**Figure 4.**
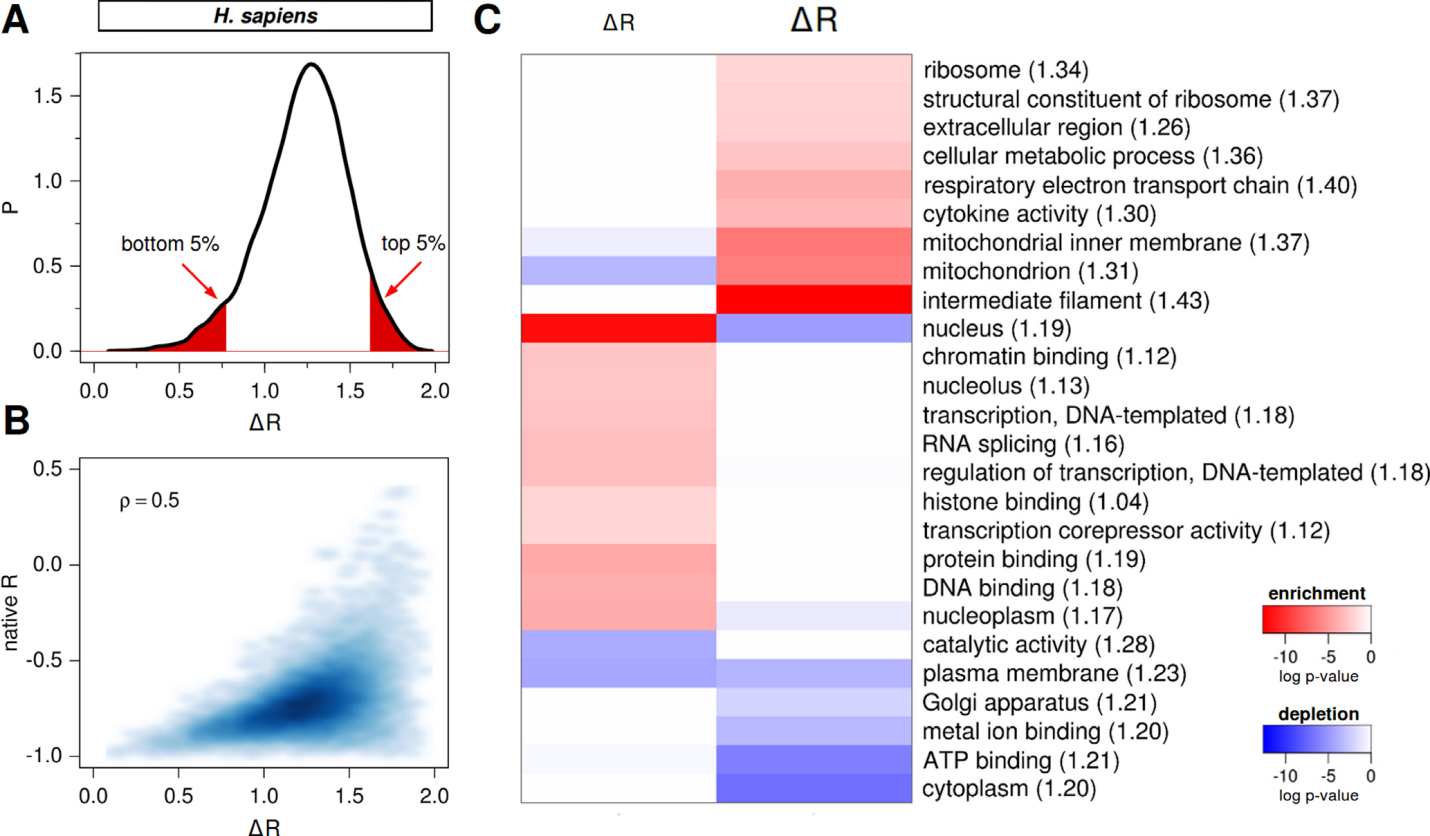
Functional significance of mRNA recoding malleability. (**A**) Distribution of the difference in Pearson *R* between the worst and the best recodings of human mRNAs as obtained by steered recoding. Regions shaded in red correspond to the bottom and the top 5% of the distribution. (**B**) Relationship between malleability (Δ*R* = *R*_worst_ – *R*_best_) and the native level of matching *R* for the human proteome quantified using Spearman correlation coefficient ρ. (**C**) A heatmap showing functional enrichment and depletion for the subset of the least (bottom 5%, left column) and the most (top 5%, right column) malleable human mRNAs, as indicated by smaller or larger Δ*R*s, respectively. The heatmap is colored based on the significance of FDR-corrected *P*-values, with red denoting enrichment and blue depletion. Numbers in the parentheses denote the average Δ*R* values per GO term over all proteins.

How malleable are different mRNAs when it comes to their potential to yield different levels of matching upon recoding? Depending on the composition of their cognate proteins, native mRNAs explore different ranges of profile matching with their cognate proteins when recoded. For each protein, we have analyzed the difference in Pearson *R* between its worst-matched and its best-matched recoded mRNA variant (Δ*R* = *R*_worst_ – *R*_best_) as a quantitative measure of malleability in complementary matching. Indeed, while the matching of a typical human protein with its cognate mRNAs covers a range of approximately 1.2 Pearson units, for some human proteins this is close to 0 (no malleability in matching) and for others close to 2 (full malleability, ranging from perfect matching to perfect anti-matching) as shown in Figure 4A. Could this potential to yield different levels of matching, or lack thereof, be biologically and functionally relevant? To address this question, we have focused on the bottom and the top 5% of the distribution of Δ*R*, that is, the extremes that exhibit the smallest and the largest changes in the level of matching upon recoding, respectively, and analyzed their GO fingerprints (Figure 4A and C). Among the least malleable human mRNAs, we observe a significant enrichment of nuclear proteins and functions related to DNA and chromatin binding, transcription and RNA splicing, while the most malleable mRNAs are completely distinct from this and encompass cytoskeletal, mitochondrial, extracellular and ribosome-related functions and processes. Interestingly, the ribosome-related functions are also strongly enriched in the group of highly malleable mRNAs in *M. jannaschii* and *E. coli* proteomes (Supplementary Tables S1 and S2). On the other hand, these two organisms exhibit no statistically significant enrichment when it comes to least malleable mRNAs.

We have also calculated the average Δ*R* for each GO term in *H. sapiens* associated with 100 or more annotated proteins (Supplementary Table S3, left) and found that GO terms enriched among the bottom 5% (Figure 4A and C) exhibit systematically lower malleabilities (Δ*R* < 1.2) than those enriched among the top 5% (Δ*R* ∼ 1.3 or greater), even over all proteins. We next analyzed the distributions of Δ*R*s for two exemplary GO molecular functions: ‘chromatin binding’ and ‘structural constituent of ribosome’, which are enriched in bottom and top 5%, respectively (Figure 4C), and showed they are significantly different (Wilcoxon rank-sum test, *P* < 2.2e-16, Supplementary Figure S2). Taken together, we conclude that even individual GO terms (Supplementary Table S3) can be distinguished based on their average Δ*R*s, further reinforcing the observation that malleability of complementary matching is associated with biological function. Interestingly, there appears to be a peculiar relationship between the malleability in matching of a given mRNA and its native level of matching (Figure 4B for *H. sapiens* and Supplementary Figure S3A and SB for *M. jannaschii* and *E. coli* transcriptomes, respectively). Overall, the lower the malleability, the higher the native level of matching: in fact, all mRNAs with Δ*R* < 0.5 have native values of *R* < −0.8. The converse, however, is not true: mRNAs with high native matching cover the whole range of Δ*R*s. On the other hand, the higher the malleability, the wider the range of native levels of matching, while the lower the native matching, the higher the malleability (Figure 4B, Supplementary Figure S3A and SB).

**Figure 5.**
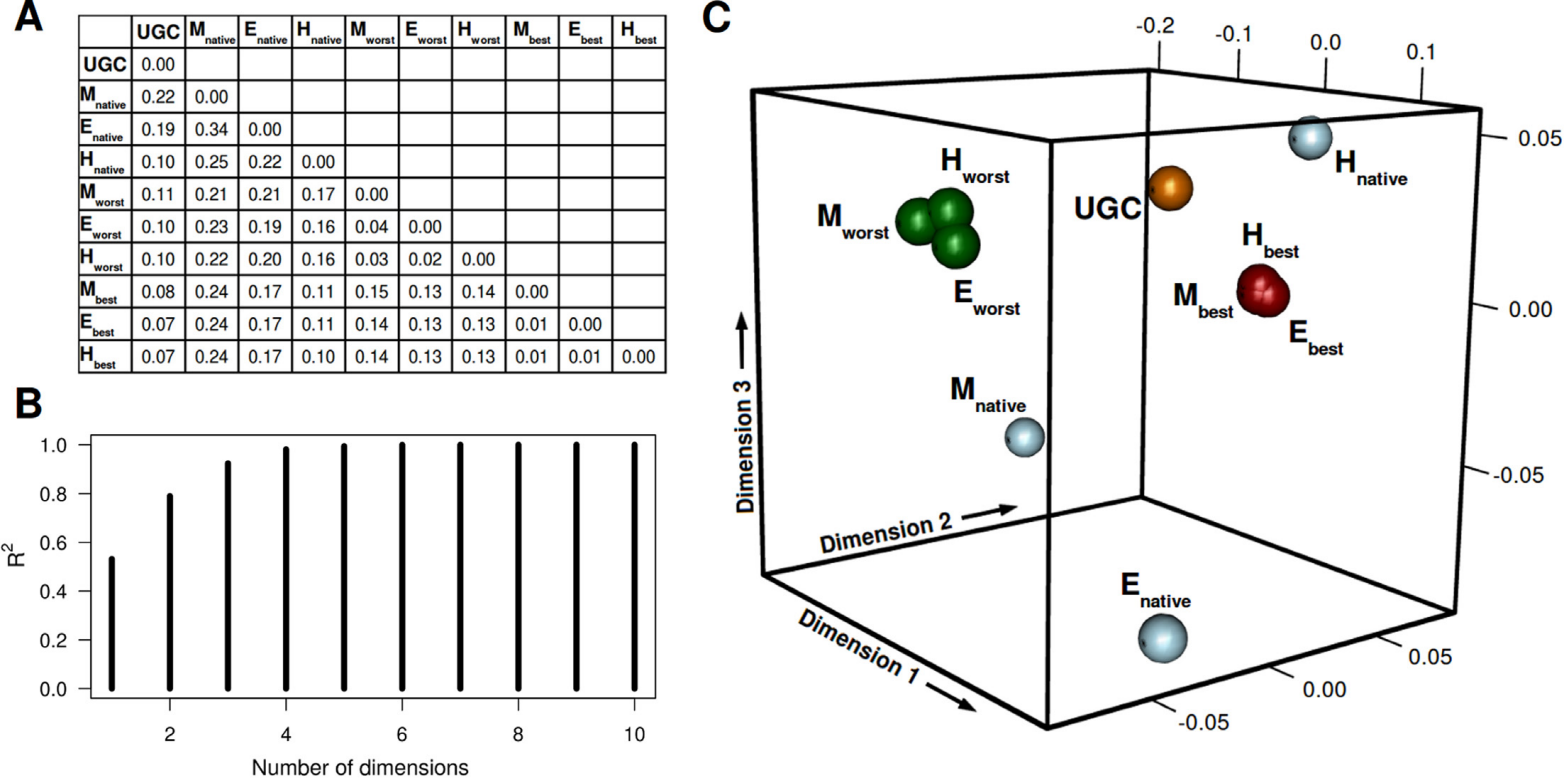
Codon-usage pattern for native and recoded human transcripts. (**A**) RMSD values between codon usage patterns corresponding to the universal genetic code (UGC) as well as native (subscript native), best-matched (subscript best) and worst-matched (subscript worst) *M. jannaschii* (**M**), *E. coli* (**E**) and *H. sapiens* (**H**) transcriptomes. (**B**) Fraction of variance captured by multidimensional scaling as a function of the number of dimensions. (**C**) Multidimensional scaling of RMSD values between different codon-usage patterns.

We have next focused on matching-optimized transcriptomes obtained via steered recoding, but this time from the perspective of their codon-usage patterns. How different are codon-usage patterns of the best-matched and the worst-matched transcriptomes when compared to either the native patterns or those based on the genetic code alone? With this in mind, we have calculated the RMSD between different codon-usage patterns involving the three organisms (native, best-matched, worst-matched and genetic-code-based, see Methods for details) and obtained a pairwise distance matrix with 45 mutual distances in total (Figure 5A). For visualization purposes, we have performed multidimensional scaling of these distances to 3D as illustrated in Figure 5B and C. Importantly, embedding in 3D results in a highly representative picture and captures over 90% of variance among all of the distances (Figure 5B). There are several important observations one can glean from this analysis. First, all three native transcriptomes (Figure 5C, blue) are biased when compared to codon frequencies based on the genetic code alone (Figure 5C, orange), with the human transcriptome being least biased, at least when it comes to the 20 codons which were considered in this analysis (see Methods for details). Second, all recoded transcriptomes (Figure 5C, red and green) also show significant bias when compared to the genetic-code baseline. Third, recoded transcriptomes of all three organisms group together such that all the best-matched transcriptomes (Figure 5C, red) populate the same region of the codon-usage space, and the same is true of all the worst-matched transcriptomes (Figure 5C, green). The best-matched and the worst-matched groups are, however, significantly distant from each other. Finally, codon-usage patterns do not appear to be related to the level of matching in native transcriptomes, as all three native transcriptomes exhibit similar levels of matching, but highly distinct codon-usage patterns. The best-matched transcriptomes, on the other hand, exhibit both similar levels of matching as well as codon-usage patterns, and the same holds for worst-matched transcriptomes. In other words, even though recoding is initiated from different starting positions (i.e. different codon-usage patterns for the three native transcriptomes), the resulting extremes all exhibit very similar codon-usage patterns, which differ between different types of extremes (i.e. best-matched *versus* worst-matched).

**Figure 6.**
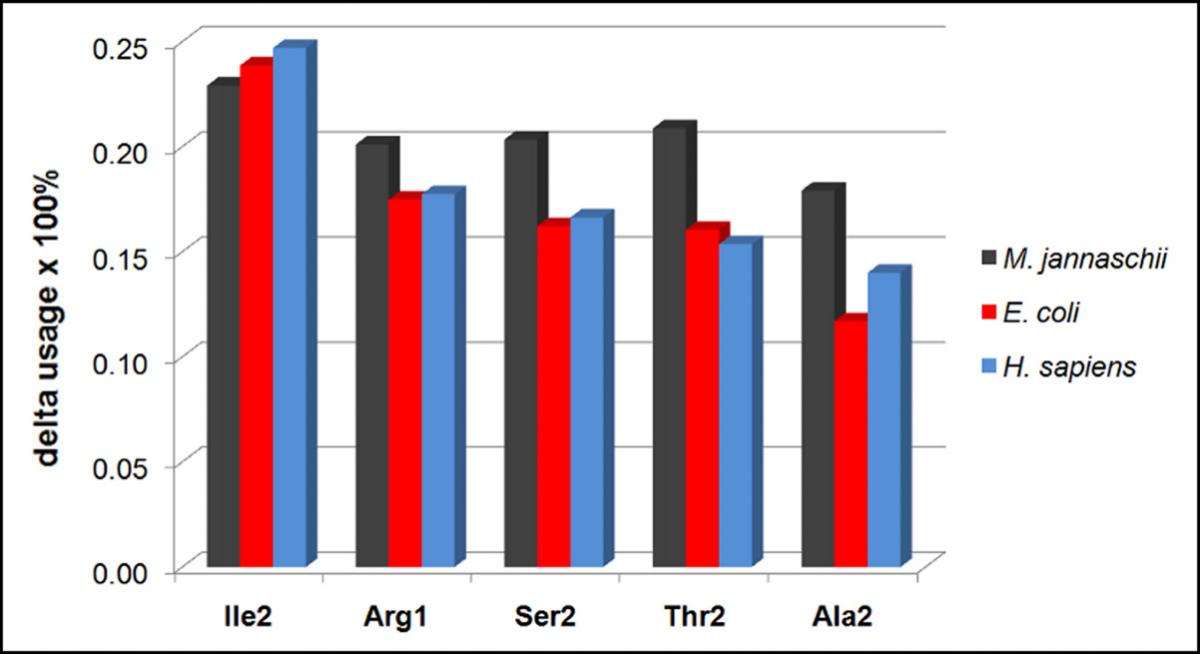
Top 5 individual codons that exhibit the largest change in their frequencies upon worst-to-best transition together with the associated relative change. The number next to the amino-acid abbreviation denotes the number of pyrimidines in the codon (e.g. Ile2 – codon for isoleucine with 2 pyrimidines etc.).

The finding that extreme transcriptomes have similar, yet highly distorted codon-usage patterns when compared to native transcriptomes, allows one to examine which particular codons exhibit the biggest change in usage upon transition from the worst- to the best-matched transcriptomes. Indeed, we observe a fairly consistent picture in all three organisms when it comes to the contribution of individual codons/amino acids to this transition (Figure 6). The top five amino acids that most significantly contribute to the shift of the profile-matching distribution upon worst-to-best transition are, starting with the most important contributor: Ile, Arg, Ser, Thr and Ala. This ranking is the same for all three organisms, with the exception of *M. jannaschii* where Arg and Thr switch places. More specifically, our analysis reveals that in order to most drastically improve the level of matching, one should increase the usage of pyrimidine-rich codons for isoleucine, threonine and alanine (Ile2, Thr2, Ala2—where the number indicates the number of pyrimidines in the codon) at the expense of their pyrimidine poor counterparts (Ile1, Thr1, Ala1). Similarly, usage of intermediate codons in terms of pyrimidine content for Arg and Ser (Arg1 and Ser2, as opposed to Arg0 and Arg2 or Ser1 and Ser3) should also increase (Figure 6). Here, it should be emphasized that codon-usage bias is by no means the only factor influencing the extent of profile matching as the amino-acid abundance and the exact amino-acid scale used for scoring protein sequences (e.g. the PR scale as used herein) also contribute significantly (1–3,6–7,10).

**Figure 7.**
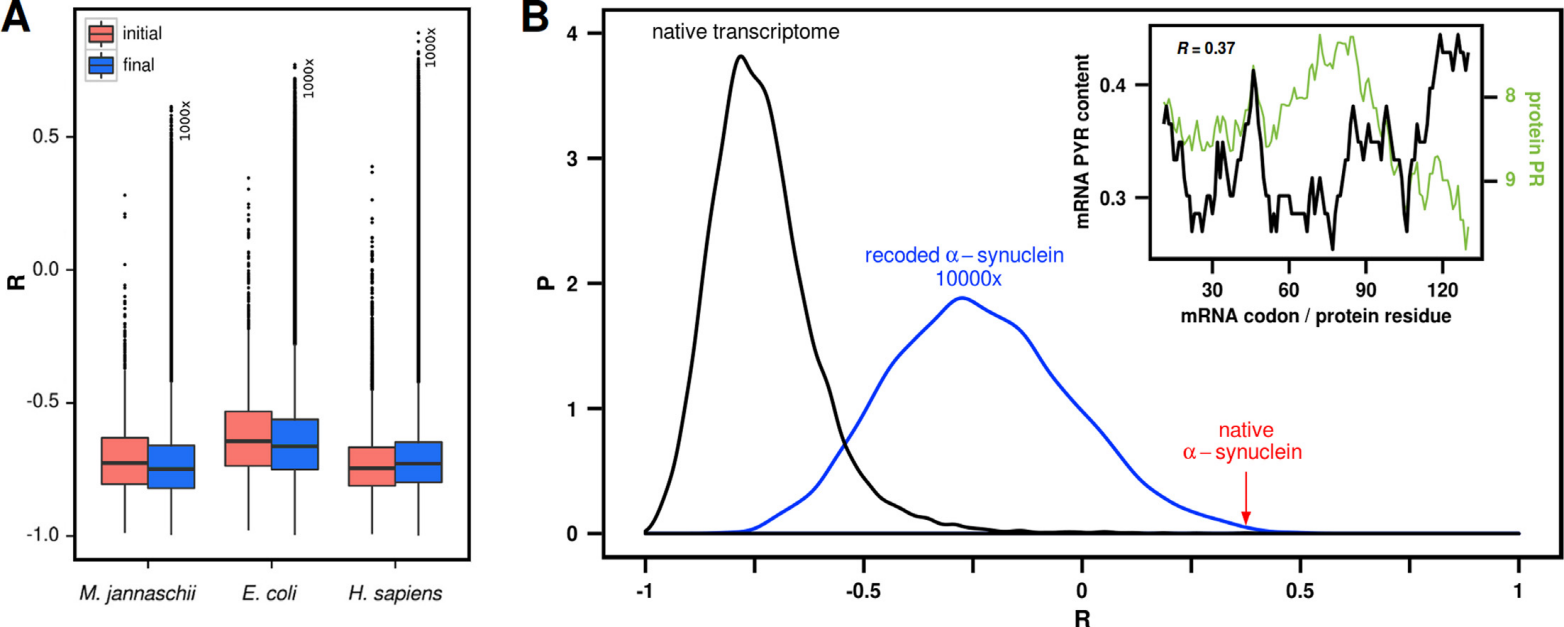
Transcriptome-wide non-steered recoding of mRNAs using codon frequencies as defined by the genetic code table. (**A**) For each organism, the initial distribution (red) captures the level of profile-matching of native transcriptomes from which non-steered recording was initiated resulting ultimately in 10 000 recoded mRNAs for each native mRNA. A representative random subsample of 1000 transcriptomes for each organism is shown in the final distribution (blue). (**B**) Distributions of profile-matching for the native human transcriptome (black) and 10 000 recoded mRNA variants of human α-synuclein (blue). Red arrow denotes the native *R* for human α-synuclein. Inset: mRNA PYR and protein PR profiles for native human α-synuclein.

Steered recoding by design results in highly biased mRNA sequences for each given set of proteins and it is not *a priori* clear what part of the accessible mRNA sequence space is covered by such extreme sequences. In fact, it is not even clear how large the fraction of the mRNA sequence space is, which yields native-like levels of matching. A highly pertinent question in this regard is what levels of cognate mRNA-protein matching would be obtained by unbiased recoding on average. In other words, what is the level of matching between a given protein and a typical random mRNA sequence belonging to it? We have performed transcriptome-wide non-steered recoding of mRNAs (see Methods for details), in which each cycle begins with the same native mRNA sequence, and finally results in 10 000 independently recoded mRNAs per native mRNA, each of them exploring the codon-usage landscape in a non-steered fashion (Figure 1, right). In this way, we have obtained an approximate, albeit undersampled picture of the complete mRNA sequence landscape, allowing us to study its global features. Our analysis reveals that mRNA sequences recoded in a non-steered fashion largely overlap with the native ones when it comes to their level of matching with cognate protein sequences (Figure 7A). For example, the average level of matching of native human sequences corresponds to a Pearson *R* of −0.73, while the grand average over 10 000 variants of the human transcriptome, recoded in non-steered fashion as described above, is −0.71 (and a similar situation is seen for *M. jannaschii* and *E. coli*). What is more, if one performs non-steered recoding of the type described above, but this time uses organism-specific codon frequencies instead of those dictated by the genetic code alone, the same conclusion is reached: native levels of matching do not require exclusive optimization to be reached, but are shared by the large majority of different realizations of synonymous transcriptomes as obtained in a non-steered manner (Supplementary Figure S4).

Non-steered recoding also allowed us to analyze how much native matching of a given mRNA/protein pair deviates from the matching of typical recoded variants of the same mRNA. For some proteins, for example, the mRNAs recoded in the non-steered fashion are on average significantly better-matched and for some they are significantly worse-matched than the native mRNA (the full list is given in Supplementary Table S4). Such signals of potential evolutionary optimization may be particularly relevant in those cases where the native level of matching is in absolute terms a significant outlier from the behavior of average proteins in a given organism. Human α-synuclein, a protein involved in the pathophysiology of Parkinson's disease, is a prime example in this sense (Figure 7B). At the Pearson *R* of 0.37, the native α-synuclein and its mRNA exhibit the second-worst level of matching among all human proteins (the only worse *R* belonging to the relatively short mitochondrial cytochrome C oxidase subunit 8, Supplementary Table S4). In addition, the native mRNA of α-synuclein is by almost three standard deviations worse-matching than its average synonymous variant recoded in a non-steered fashion (Figure 7B, Supplementary Table S4). In other words, the native mRNA of α-synuclein appears to be significantly optimized to weaken compositional complementarity with its cognate protein, and one may speculate that the purpose of this may be to reduce the binding between the two, whatever its potential biological function may be. What makes this particularly attractive is the fact that α-synuclein exhibits a strong propensity to self-aggregate in the cell (25–27). It is possible that complementary interactions with their cognate mRNAs help solubilize proteins, a feature which may be reduced in the case of α-synuclein and its mRNA for some other biological purpose. Recently, Tartaglia and coworkers have used computational methods to predict that α-synuclein may strongly interact with its cognate transcript, especially in the 5′ UTR (28). It will be exciting to explore a potential connection between these two not necessarily contradictory findings. The above possibilities notwithstanding, our preliminary GO analysis of mRNA/protein pairs with native sequences that appear particularly well-optimized for or against matching (outliers in the list given in Supplementary Table S4) did not reveal any statistically significant enrichment of particular functions or processes among these two groups (data not shown).

## DISCUSSION

The level of compositional sequence complementarity between a given mRNA and its cognate protein depends, in principle, on three key factors: (1) the structure of the genetic code, (2) the specific composition of the mRNA and (3) the specific composition of the protein. Changes in any of these three factors could affect the level of complementary mRNA/protein matching. Here, we have asked how synonymous mRNA recoding affects the level of matching with its cognate protein, provided that the universal genetic code is used for encoding and that protein sequence remains fixed. Remarkably, we have shown that it is indeed possible to recode the mRNA pools of different organisms such that the level of matching with their cognate proteins is not only nullified, but actually moved in the direction of significant anti-matching on average (Figures 2 and 3). Importantly, however, our results show that the great majority of possible mRNA encodings still retain the native-like level of matching, with only a small subset of mRNA sequences having the potential to either strongly improve or worsen the native matching (Figure 7A). In light of the hypothesis that compositional complementarity between mRNAs and their cognate proteins is indicative of their binding potential (1–3,6–7,10), our results suggest that although most mRNA variants for a given protein exhibit similar binding potential, the genetic code provides enough flexibility to steer it in any direction needed.

There are several caveats concerning our analysis that should be discussed. First, we have used the standard genetic code throughout our randomization experiments, which may have biased the results in the case of eukaryotic proteins encoded by the mitochondrial genome as these are known to be translated via a modified genetic code. However, given that there are only 13 such proteins in the human mitochondrial genome (29), this has not affected our main conclusions to any appreciable extent. Second, there is no guarantee that the PR scale (5), which was in an invariant form used in all of the above analysis, optimally reflects the physical interaction propensity of different amino acids for real codons. This scale, based on pyridine interaction propensity of individual amino acids (1,4–5), is only a proxy for their interaction propensity with uracils and cytosines. It is possible that the malleability in terms of codon-usage patterns as discussed above is biased by the properties of the PR scale used. On the other hand, the general consistency between the PR scale and knowledge-based potentials capturing the affinity of amino-acid residues for biologically relevant pyrimidines (3) suggests that the bulk of the effect is still captured by the PR scale. In any case, it will be important to perform similar analyses using other nucleobase/amino-acid interaction propensity scales (3,6–7). Our expectation, however, is that one would see general behavior similar to that of the PR scale. Finally, all of our analysis was carried out at the primary sequence level and it is not *a priori* clear how this relates to the case of folded 3D structures of mRNAs and proteins. One possibility, already discussed before (1–3,6–7,10), is that putative complementary binding manifests itself only in those situations where both polymers are largely unstructured, such as, for example, during translation, but we do not exclude the possibility of such interactions also being relevant for folded biopolymers (10).

Our analysis has shown that there is a strong statistical association between the malleability of mRNAs and certain biological functions and processes, and it is tempting to speculate that this may indicate functional significance of profile matching for these groups of mRNAs and proteins. Indeed, low malleability is strongly associated with a high level of native matching (Figure 4B), and it is possible that in those cases, direct binding between mRNAs and their cognate proteins may play an important functional role. mRNA sequences with low malleability are indeed enriched with nuclear functions and processes, many of which involve direct interactions between nucleic acids and proteins, including cognate ones (Figure 4C). Alternatively, it is possible that the observed association between malleability and function simply reflects either compositional biases or insufficient sampling due to length differences of these particular subsets of proteins/mRNAs. It is, however, generally hard to separate and evaluate these effects independently since they are simply inherent properties of proteins/mRNAs. Larger differences between the worst-matching sets than between the native or the best-matching sets belonging to the three organisms studied herein (Figure 3) are there probably because of similar reasons. First, these differences reflect the features of proteome composition and codon usage bias, which differ between different organisms. For example, a lower abundance of residues whose codon pyrimidine content can at all be changed means less dramatic shifts no matter how good the sampling. In fact, the extent of the shifts in the worst direction upon recoding (i.e. *R*_worst_ − *R*_native_ which equals 0.9, 0.99 and 1.06 for *M. jannaschii, H. sapiens* and *E. coli*, respectively, Figure 3) follows the same order as the fraction of residues in those proteomes whose codon PYR content can at all be changed (i.e. fractions of Ile, Leu, Val, Thr, Ala, Ser, Pro, Arg, Gly combined, which are 54.3%, 59.3% and 61.9% for *M. jannaschii, H. sapiens* and *E. coli*, respectively). Second, the above difference may to some extent be a consequence of incomplete sampling: it is possible that the worst-matching distributions are not fully converged, unlike the best-matching distributions. Although both our convergence analysis (Supplementary Figure S1) as well as our analysis of codon-usage bias among the worst sequences (Figure 5) speak against this possibility, one cannot fully discount it. However, we should again emphasize that the primary goal of the present study was to test if significant shifts of matching distributions are at all possible, and not to determine them exactly.

The ability to recode entire mRNA transcriptomes to exhibit a much stronger or a much weaker level of matching when compared to native ones (Figure 3) comes at a price of distorting the original, organism-specific codon-usage bias (Figure 4C). This may provide a part of the explanation for why one does not necessarily observe the levels of matching that are as high or low as in the extreme transcriptomes obtained via steered recoding. Namely, organism-specific codon-usage bias represents an evolutionarily optimal solution to a set of different requirements and constraints. For example, it is known that codon-usage bias in a given organism mostly reflects mutational biases (i.e. biases in point mutations, or biases during base repair) or the adaptation to cellular tRNA abundances (i.e. translational optimization) to various extents (11–12,30–31). In addition, it may also directly influence mRNA secondary structure (32,33), or even the efficiency of transcription factor binding to coding regions of DNA (34). The fact that different organisms exhibit distinct codon-usage signatures actually reflects the complexity of meeting various specific requirements when it comes to gene regulation and expression in an organism-specific manner. On the other hand, the level of mRNA-protein complementarity appears to be largely insensitive to details of organism-specific codon-usage bias; in fact, the large majority of different mRNA encodings for most proteins lead to significant levels of complementary matching (Figure 7A). We would like to interpret this as evidence that such complementarity is or was indeed functionally relevant to such a degree that it was actually embedded in the structure of the genetic code. As discussed before (1–3,6–7,10), one such function in primordial systems could have been translation via direct templating of proteins from their cognate mRNAs, which as a corollary could have led to complementarity between the two polymers. However, from structural stabilization of mRNAs to translational feedback control to protein and mRNA chaperone activity (2), cognate mRNA/protein interactions could be functionally relevant even in present-day systems and even to a degree that is still not fully appreciated. Future work will shed light on this exciting possibility.

## SUPPLEMENTARY DATA

Supplementary Data are available at NAR Online.

SUPPLEMENTARY DATA
