# Supplementary material for: Malleable nature of mRNA-protein compositional complementarity and its functional significance
Source: Nucleic Acids Res. 2015 Mar 8;43(6):3012–21. doi: 10.1093/nar/gkv166 (PMC4381073; doi:10.1093/nar/gkv166)
Supplement: SUPPLEMENTARY DATA [file supp_43_6_3012__index.html]

Malleable nature of mRNA-protein compositional complementarity and its functional significance — Malleable nature of mRNA-protein compositional complementarity and its functional significance — Malleable nature of mRNA-protein compositional complementarity and its functional significance — SUPPLEMENTARY DATA 

# Malleable nature of mRNA-protein compositional complementarity and its functional significance

## SUPPLEMENTARY DATA

**Files in this Data Supplement:**

- SUPPLEMENTARY DATA
- SUPPLEMENTARY DATA
