# Supplementary material for: Malleable nature of mRNA-protein compositional complementarity and its functional significance
Source: Nucleic Acids Res. 2015 Mar 8;43(6):3012–21. doi: 10.1093/nar/gkv166 (PMC4381073; doi:10.1093/nar/gkv166)
Supplement: SUPPLEMENTARY DATA [file supp_gkv166_nar-00134-n-2015-File009.doc]

**Supplementary Data**

for the manuscript

**Malleable nature of mRNA-protein compositional complementarity and its functional significance**

Mario Hlevnjak & Bojan Zagrovic*

Department of Structural and Computational Biology

Max F. Perutz Laboratories & University of Vienna, Vienna Campus Biocenter 5, 1030 Vienna, Austria

^*^to whom correspondence should be addressed. Tel: +43-1-4277-9522; Fax: +43-1-4277-9522; Email: bojan.zagrovic@univie.ac.at

**
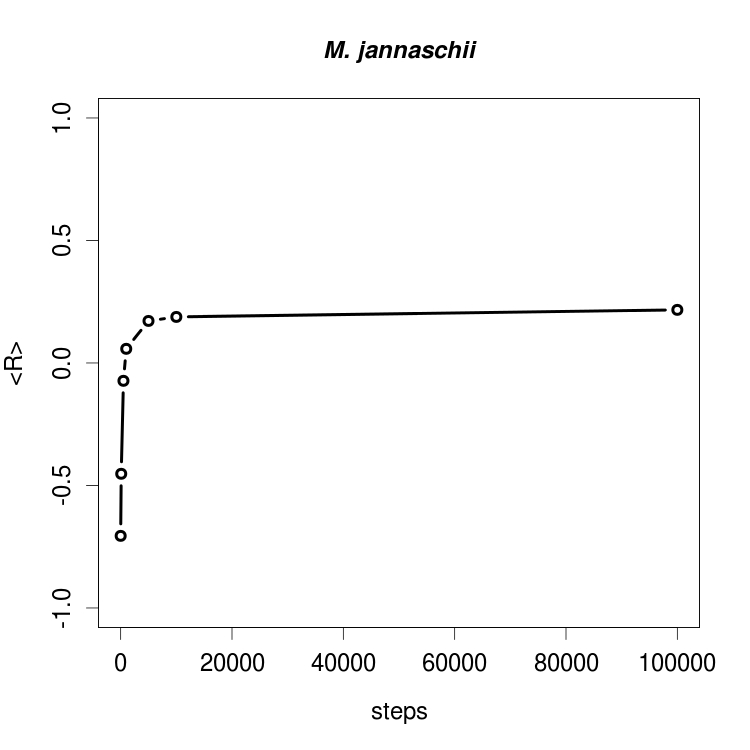
**

**Figure S1.** mRNA-trancriptome-wide average Pearson *R* as a function of the number of recoding steps for *M. jannaschii* transcriptome when optimized towards worst profile-matching using steered recoding procedure.


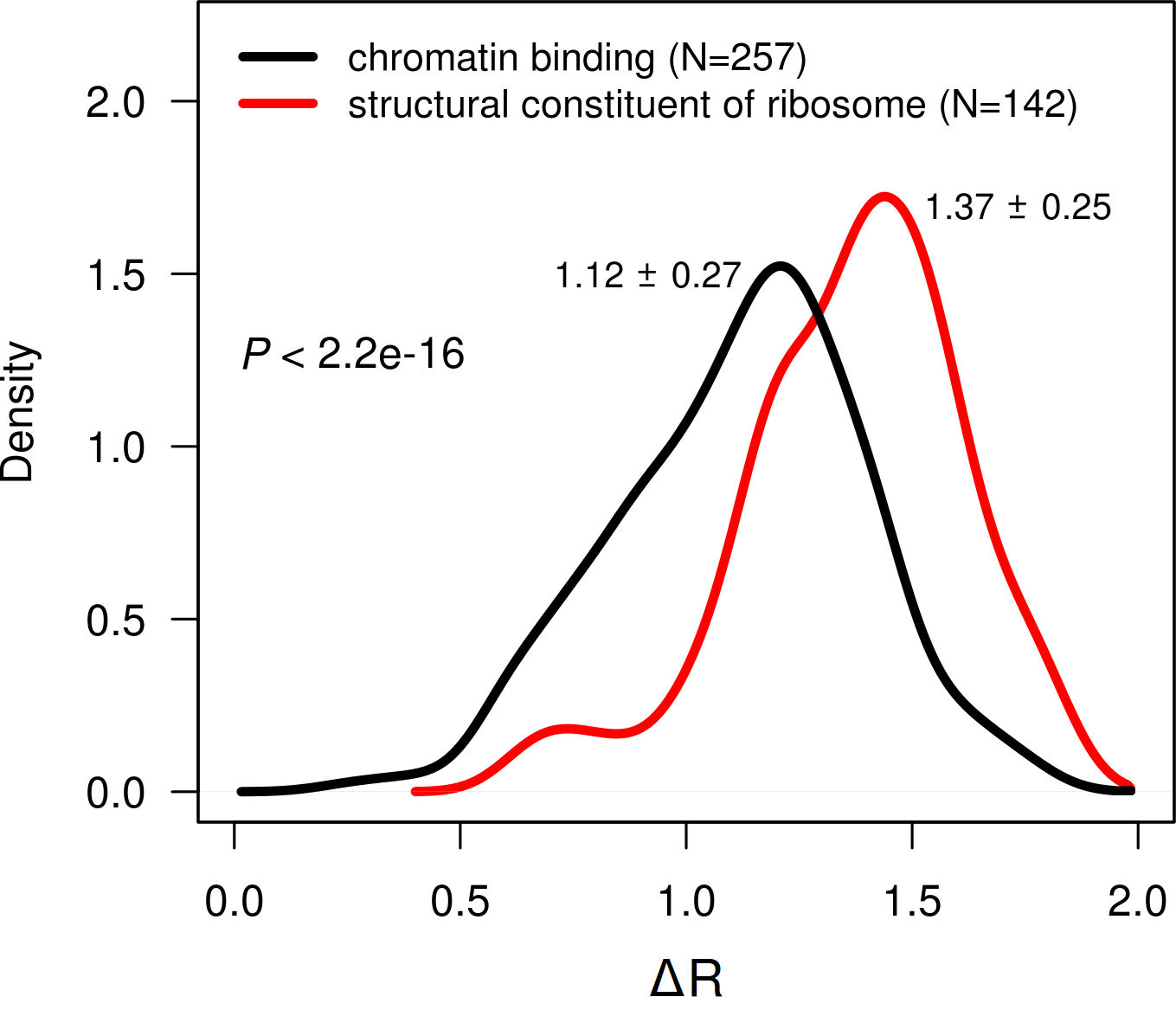


**Figure S2.** Distributions of malleabilities (Δ*R*s) for two exemplary GO molecular functions: GO:0003682 "chromatin binding" and GO:0003735 "structural constituent of ribosome" which are significantly different (Wilcoxon rank-sum test, *P* < 2.2e-16). Shown are means +/- standard deviations of Δ*R*s. N denotes the number of proteins per GO term.


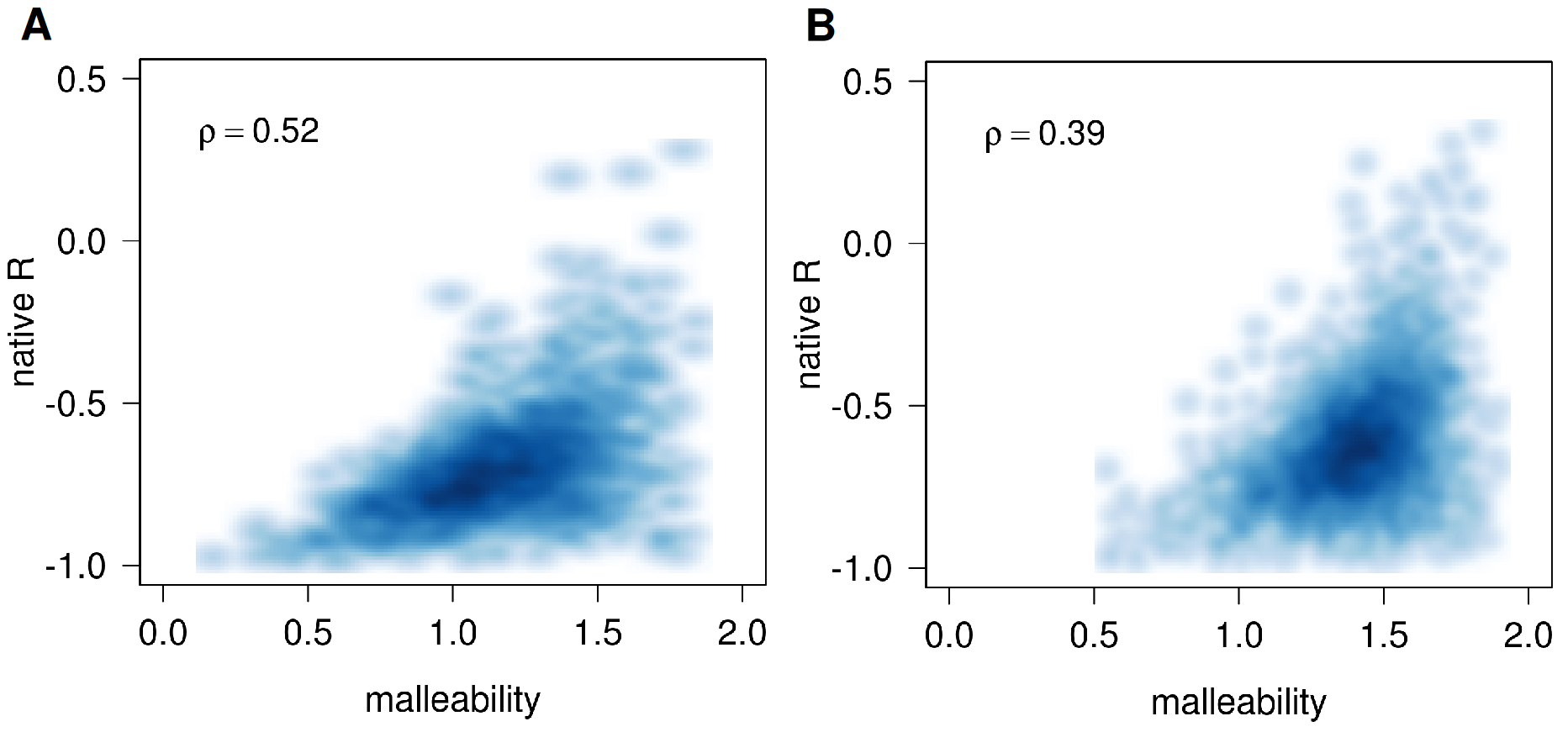


**Figure S3.** Relationship between malleability (Δ*R* = *R*_worst_ – *R*_best_) and the native level of matching *R* for A) *M. jannaschii* and B) *E. coli* proteomes. The strength of association is quantified using Spearman correlation coefficient ρ.


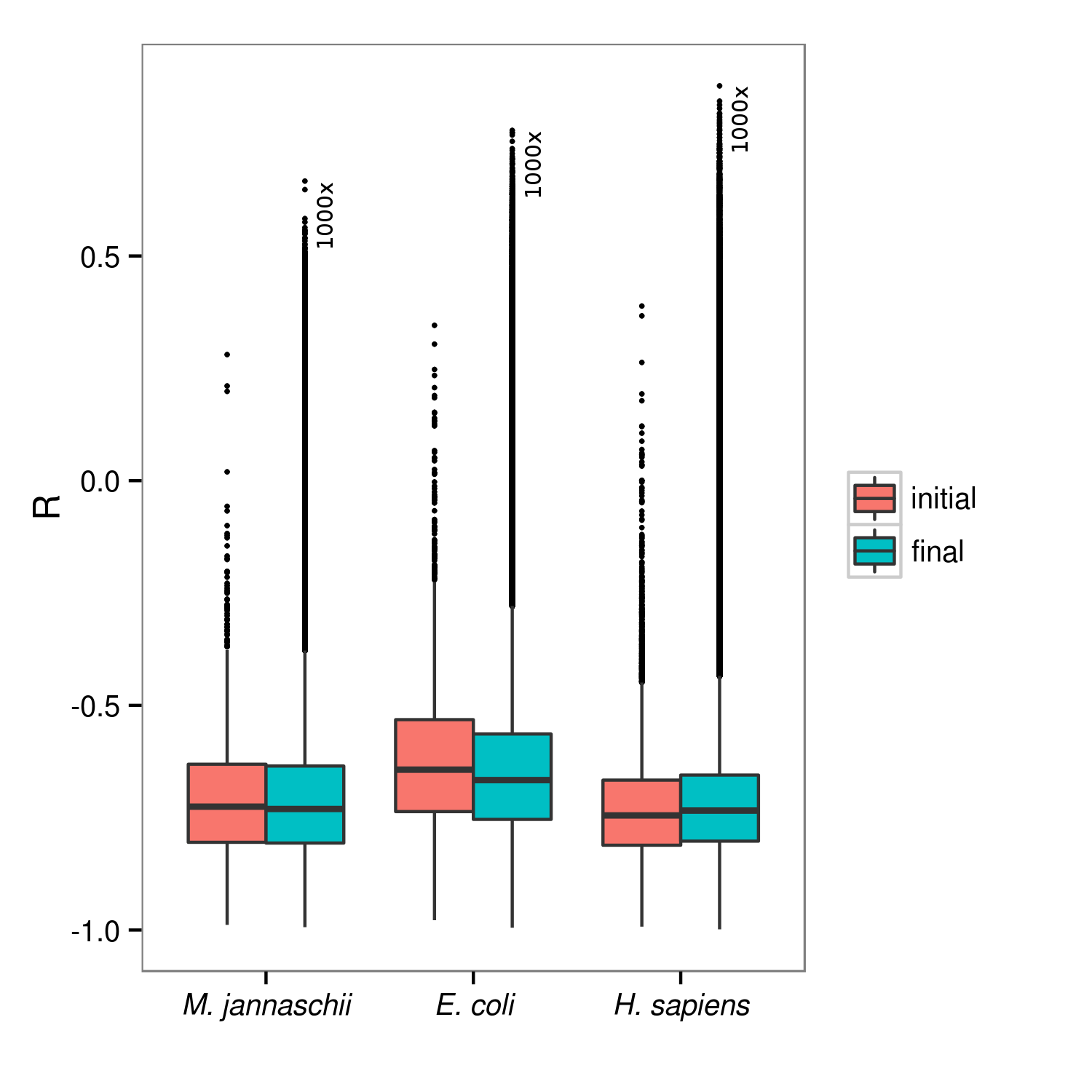


**Figure S4.** mRNA-transcriptome-wide non-steered recoding of mRNAs using organism-specific codon occurrences. For each organism, initial distribution (red) captures the level of profile-matching of native transcriptomes from which non-steered recording was initiated resulting ultimately in 1000 recoded mRNAs for each native mRNA, all of which are included in the final distribution (blue).
